# Donor NKG2C Copy Number: An Independent Predictor for CMV Reactivation After Double Cord Blood Transplantation

**DOI:** 10.3389/fimmu.2018.02444

**Published:** 2018-10-23

**Authors:** Kai Cao, David Marin, Takuye Sekine, Gabriela Rondon, Weicheng Zhao, Nathaniel T. Smith, May Daher, Qing Wang, Li Li, Rima M. Saliba, Ravi Pingali, Uday Popat, Chitra Hosing, Amanda Olson, Betul Oran, Rafet Basar, Rohtesh S. Mehta, Richard Champlin, Elizabeth J. Shpall, Katayoun Rezvani

**Affiliations:** ^1^Department of Laboratory Medicine, MD Anderson Cancer Center, Houston, TX, United States; ^2^Stem Cell Transplantation and Cellular Therapy, MD Anderson Cancer Center, Houston, TX, United States

**Keywords:** NK cells, CBT, NKG2C, CMV reactivation, graft selection

## Abstract

Cytomegalovirus (CMV) remains a major cause of morbidity following allogeneic hematopoietic stem cell transplant. Natural killer cells expressing *NKG2C* have been shown to play a role in the immune surveillance of human CMV. We studied *NKG2C* copy number in the donor graft and the risk of CMV reactivation after double umbilical cord blood transplantation (DUCBT) in 100 CMV seropositive DUCBT recipients and their corresponding cord blood (CB) grafts (*n* = 200). In the setting of DUCBT, the combined graft may contain 0–4 functional copies of *NKG2C* gene. Sixteen patients received a combined graft with 1 or 2 *NKG2C* copies and 84 patients were recipients of a combined graft with 3 or 4 *NKG2C* copies. The 6-month cumulative incidence of CMV reactivation for the two groups was 93.7 and 58.4%, respectively (*p* = 0.0003). In multivariate analysis, low *NKG2C* copies in the graft was an independent predictor of CMV reactivation (HR = 2.72, CI = 1.59–4.64; *p* < 0.0001). Our study points to an important role for donor *NKG2C* for protection against CMV reactivation after DUCBT. These novel findings may help identify patients at a higher risk of CMV reactivation after DUCBT. Donor *NKG2C* genotype may be used as a potential criterion in the algorithm for graft selection for DUCBT.

## Key points

NKG2C copy number in the CB graft is an independent risk factor for CMV reactivation after double CB transplantation.

## Introduction

Cytomegalovirus (CMV) is a member of the herpesviridae family and remains latent in the host cells after primary infection. The virus reactivates in the immunosuppressed host and is a leading cause of morbidity following allogeneic hematopoietic stem cell transplant (allo-HSCT) (1–3). Despite available therapeutic agents and preemptive approaches, CMV associated complications and end-organ damage remain a major cause for concern following allo-HSCT (4, 5).

Natural killer (NK) cells are a subset of effector lymphocytes involved in innate immunity. Unlike T cells, NK cells do not need prior antigen sensitization to kill their target cells and hence provide the first-line of defense against viruses (6). Natural killer (NK) cells are also known to be the first subset of lymphocytes to reconstitute after HSCT and hence play an important role in the early control of viral infections including CMV (7). Evidence for an NK cell response against CMV comes from murine studies (8, 9), and human studies reporting preferential expansion of NKG2C-expressing NK cells in CMV-infected individuals (10, 11). In the first year following allo-HSCT and cord blood (CB) transplantation, CMV reactivation induces expansion of a subset of mature “adaptive” NK cells, characterized as CD56^dim^CD16^−^NKG2C^+^ (12–14). Taken together, these data point to a potential role for NKG2C in CMV recognition by NK cells.

NKG2C is an activating receptor that recognizes HLA-E in complex with leader sequence peptides (15). The gene encoding NKG2C (*killer-cell lectin-like receptor C2, KLRC2)* is present at different copy numbers in the genomes of different individuals (16, 17). A homozygous deletion of the *NKG2C* gene (*del/del*) has been reported in up to 4% of the population, while up to 34% of the European and Japanese population have a heterozygous deletion (*wt*/*del*) (16, 18). *NKG2C* deletion has been reported as a risk factor for CMV (19) and HIV (20), but current data on the influence of *NKG2C* gene-copy number variations in the donor graft on the risk of CMV reactivation after allo-HSCT is limited. Following CBT, where the T-cell compartment is functionally naïve, the *NKG2C* genotype may have an even more pronounced impact on the risk of CMV reactivation. Thus, we studied *NKG2C* copy numbers in the donor graft and the risk of CMV reactivation in recipients of double umbilical cord blood transplantation (DUCBT).

## Methods

### Study design

This retrospective study was designed to test the hypothesis that lower NKG2C copy number in the CB graft is associated with a higher risk of CMV reactivation after DUCBT. *NKG2C* genotyping was performed on genomic DNA collected from 200 CB donor grafts that were infused into 100 DUCBT recipients. The *NKG2C* gene copy number was assessed by PCR-SSP (17) as described below, based on the availability of specimen, and without preference given to patients with particular clinical characteristics.

### Patients

All CMV-seropositive patients who received a DUCBT for the treatment of hematologic malignancies at MD Anderson Cancer Center between 7/2005 and 12/2012 were included. All patients consented to the study in accord with the Declaration of Helsinki, and local ethics approval was obtained before sample collection. Patient characteristics are described in Table 1.

**Table 1:** Patients characteristics and 6-month CMV reactivation rate (*n* = 100).

	***n***	**HR, (95%CI)**
Age^a^		*p* = 0.34
≤ 40 year	48	1
>40 year	52	1.26 (0.78–2.04)
Sex		*p* = 0.24
Male	43	1
Female	57	1.34(0.82–2.19)
Diagnosis		*p* = 0.86
Acute lymphoblastic leukemia	22	1
Myeloid malignancies^b^	61	0.93(0.53–1.63)
Lymphoid malignancies^c^	17	0.78 (0.33–1.88)
Disease status at transplant		*p* = 0.71
Complete remission	55	1
Relapsed/refractory disease	45	0.91
Disease risk index^d^		*p* = 0.82
Low	5	1.00 (0.26–3.84)
Intermediate	29	1
High	41	1.02 (0.59–1.77)
Very high	20	0.74 (0.37–1.50)
Conditioning regimen		*p* = 0.14
Myeloablative	20	1
Reduced intensity	66	0.62 (0.385–1.01)
Non-myeloablative	14	0.93 (0.38–2.26)
ATG treatment		*p* = 0.40
No	17	1
Yes	83	1.26 (0.73–2.18)
HLA match between recipient and dominant CB unit^e^		*p* = 0.20
7–8/8	12	1
5–6/8	39	0.51 (0.21–1.24)
≤ 4/8	38	0.75 (0.30–1.87)

a *The median age was 43 (range 7–73)*.

b *Forty-five patients had acute myeloid leukemia, 6 patients had secondary acute myeloid leukemia and 10 patients had myelodysplastic syndrome*.

c *Two patients had Hodgkin lymphoma, 5 patients had chronic lymphocytic leukemia, 9 patients had non-Hodgkin lymphoma and 1 patient had multiple myeloma*.

d *Five patients had missing data*.

e *Eleven patients had mixed chimerism, therefore the dominant unit could not be determined*.

Cord unit dominance, achieved by most patients, was defined as the unit with >90% chimerism in the total DNA fraction at the time the assay was performed.

Peripheral blood was collected twice a week for CMV monitoring. The median time to CMV reactivation was 1.4-months and the 6-month cumulative incidence of CMV reactivation was 63.3%.

### *NKG2C* genotyping by PCR amplification with sequence-specific primers (PCR-SSP)

*NKG2C* gene copy numbers were assessed by PCR-SSP (17). Two pairs of primers were used to detect the NKG2C genotypes. Primer NKG2C/F (5′-CAGTGTGGATCTTCAATG-3′) and NKG2C/R (5′-TTTAGTAATTGTGTGCATCCTA-3′) amplify a 201 bp fragment from *NKG2C* wild type (wt) carrier. Primer NKG2Cdel/F (5′-ACTCGGATTTCTATTTGATGC-3′) and NKG2Cdel/R (5′-ACAAGTGATGTATAAGAAAAAG-3′) amplify a 411 bp fragment from *NKG2C* deletion (del) carrier. A single-tube PCR-SSP genotyping strategy combining the two sets of primers was validated and modified from the method by Moraru et al. (17). Briefly, genomic DNA sample was mixed with the two sets of primers at a final concentration of 1 μM for NKGC2/F and NKGC2/R, and 0.5 μM for NKG2Cdel/F, and NKG2Cdel/R, dNTPs, PCR buffer, Taq polymerase, and PCR amplified under the following thermal cycling conditions: 1 cycle of 2 min at 95°C, then 10 cycles of 20 s at 95°C, 30 s at 60°C, and 40 s at 72°C, and 20 cycles of 20 s at 95°C, 30 s at 56°C, and 40 s at 72°C. The last extension cycle of 3 min at 72°C was followed. The PCR product was visualized using gel electrophoresis and UV exposure (Supplementary Figure 1).

### Statistical methods

The probability of CMV reactivation was calculated using the cumulative incidence method. Univariate analysis was performed with standard statistical methodology. Variables found to be significant at the *p* < 0.15 level were included in the multivariate Fine-Gray regression analysis. Hazard ratios (HR) are reported with 95% confidence intervals (CI). All *p*-values are two-sided.

## Results

### *NKG2C* copy number in the CB grafts predicts for CMV reactivation after DUCBT

Individuals may inherit different copy numbers of the *NKG2C* genes (0, 1, or 2). In our cohort, ~2/3 of the CB units had both copies of the gene (*wt/wt*), 1/3 had only one copy (*wt/del*), and only a minority of units had 0 copies with both alleles deleted (*del/del*, Table 2). Since all patients received a DUCBT, the combined graft could contain from 0 to 4 functional copies of the *NKG2C* gene. Patients whose combined grafts contained only 1 or 2 *NKG2C* gene copies (i.e., *wt/del* and *wt/del*, or *del/del* and *wt/wt*, or *wt/del* and *del/del*) had a significantly higher probability of CMV reactivation than patients whose combined grafts had 3 or 4 *NKG2C* copies (i.e., *wt/wt* and *wt/del* or *wt/wt* and *wt/wt*). Namely, the 6-month cumulative incidence of CMV reactivation for the four groups was 100, 92.9, 60.5, and 55.9%, respectively (*p* = 0.005). No patient received a graft with zero gene copies.

**Table 2:** NKG2C genotype of the CB units and 6-months CMV reactivation rate (*n* = 100).

	***n***	**HR, (95%CI)**
NKG2C genotype of CB unit 1		*p* = 0.81
wt-wt (2 copies)	66	1
wt-del (1 copy)	31	1.18 (0.71–1.98)
del-del (0 copies)	3	1.16 (0.28–4.85)
NKG2C genotype of CB unit 2		*p* = 0.16
wt-wt (2 copies)	64	1
wt-del (1 copy)	32	1.24 (0.61–2.48)
del-del (0 copies)	4	2.95 (0.99–5.80)
NKG2C genotype of the dominant CB unit^a^		*p* = 0.51
wt-wt (2 copies)	59	1
wt-del (1 copy)	30	1.19 (0.71–1.99)
del-del (0 copies)	0	–
NKG2C copy number in the combined CB units		*p* = 0.005
4	41	1
3	43	1.01 (0.58–1.77)
2	14	2.65 (1.37–5.09)
1	2	2.49 (0.76–8.16)
0	0	–
NKG2C copy number in the combined cord units		*p* = 0.0003
3–4	84	1
1–2	16	2.61 (1.54–4.41)

a *Eleven patients had mixed chimerism, therefore the dominant unit could not be determined*.

**Figure 1:**
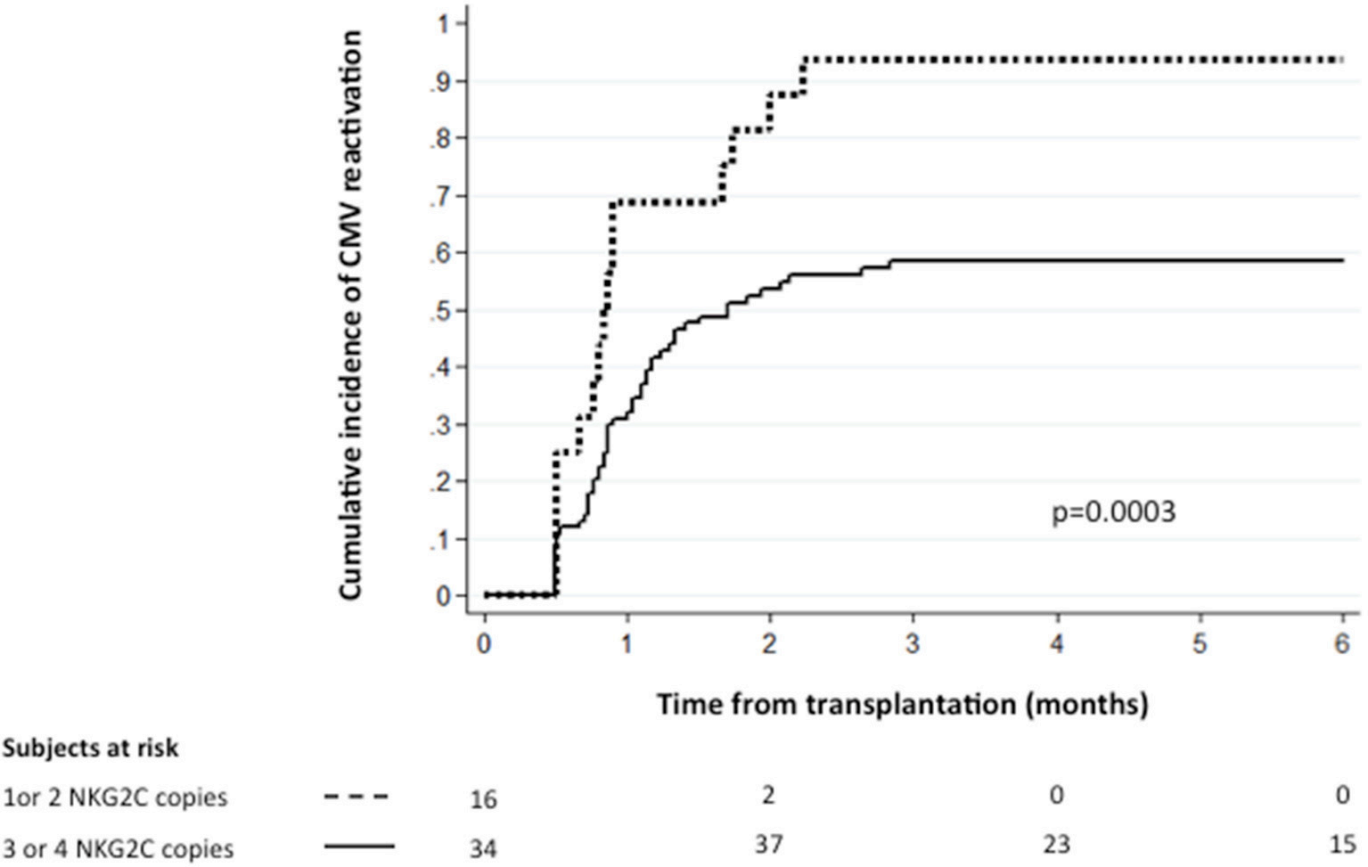
The cumulative incidence of CMV reactivation following DUCBT stratified by NKG2C copy number. Graft *NKG2C* copy number and the conditioning regimen intensity are the only independent predictors for CMV reactivation after DUCBT.

For the rest of the analysis we divided the patients into two groups, namely the 84 patients who received two CB grafts with 3 or 4 *NKG2C* copies and the 16 patients who received two units with 1 or 2 *NKG2C* copies. Notably, the clinical characteristics (as defined in Table 1) for the two groups of patients were similar (data not shown). The 6-month cumulative incidence of CMV reactivation for the two groups was 58.4 and 93.7%, respectively (*p* = 0.0003, Figure 1). Interestingly, the *NKG2C* copy number of the dominant cord was not predictive for CMV reactivation, suggesting that both cord units contribute to the antiviral response early post-DUCBT (Table 2).

We performed univariate and multivariate analysis that included *NKG2C* copy number and the variables shown in Table 1. Low *NKG2C* copy number (1 or 2) in the combined CB graft (HR = 2.72, CI = 1.59–4.64; *p* < 0.0001) and reduced intensity conditioning (RIC; HR = 0.59, CI = 0.35–0.99, *p* = 0.046) were the only independent predictors for CMV reactivation. We also examined the influence of acute graft vs. host disease (aGVHD) on the risk of CMV reactivation using a time-dependent regression model. The development of grade II–IV or III–IV aGVHD did not significantly impact the probability of CMV reactivation (data not shown). This could be explained by the fact that most patients developed CMV reactivation prior to aGVHD onset. The 2-month cumulative incidence of CMV reactivation was 57.0% while the 2-month cumulative incidence of grade III-IV aGVHD was 7.0%. A number of studies have reported a protective effect of CMV reactivation on relapse (21, 22). However, we found no significant impact of graft *NKG2C* copy number on the risk of relapse after DUCBT (data not shown).

## Discussion

Conventionally NKG2C-expressing NK cells have been linked to efficient recognition and elimination of CMV-infected cells (12, 13). This observation, combined with studies reporting that the frequencies and regulation of NKG2C receptor expression is dependent on the gene copy number (19, 23), led us to hypothesize that the risk of CMV reactivation after DUCBT could be directly influenced by the *NKG2*C copy number of the CB grafts.

To our knowledge our study is the first to assess the influence of NKG2C genotype in both CB units on CMV reactivation in the setting of DUCBT. We extensively analyzed all possible genotype combinations including dominance of engraftments looking at the distinct incidence rates of CMV reactivation, which had not previously been done.

Although the number of patients analyzed in this study is limited, our data point to an association between *NKG2C* gene copy number in the CB grafts and the risk of CMV reactivation after DUCBT. We show that recipients of two grafts with a *wt/del* and *wt/del*, or *wt/wt* and *del/del* genotype are inherently more susceptible to CMV reactivation, with the vast majority of patients developing CMV reactivation in the first 3-months post-CBT. This effect is likely related to quantitative differences in NKG2C expression on reconstituting NK cells after CBT. Although our study cohort did not include a case with both grafts carrying the *del/del* genotype, our results are in agreement with a previous study reporting CMV reactivation in three patients receiving a single CB graft with the *NKG2C del/del* genotype (24). Our multivariate analysis also shows that the only two independent predictors of CMV reactivation after DUCBT are low NKG2C copy number in the combined graft and reduced intensity conditioning regimen. The latter is in keeping with previous reports of better outcomes following RIC for DUCBT (25–27).

In summary, our results point to an important role for NKG2C in protection against CMV after CBT. Further studies are warranted to understand the precise mechanistic role of NKG2C+ NK cells in CMV recognition. If confirmed in larger numbers of CBT recipients, *NKG2C* genotyping of the CB graft may be a useful biomarker for predicting the risk of CMV infection after CBT, thus, guiding the intensity of CMV prophylaxis for individual patients. Moreover, it may provide a compelling rationale for considering *NKG2C* genotype in the algorithm of CB selection.

## Author contributions

KC performed experiments, designed, interpreted, analyzed, and commented on the manuscript. DM interpreted, analyzed, and wrote the manuscript. TS, WZ, NS, MD, QW, RB, and LL performed experiments, analyzed, and commented on the manuscript. GR, RS, RP, UP, CH, AO, BO, RM, RC, and ES collected clinical data, interpreted, analyzed, and commented on the manuscript. KR designed and directed the study and wrote the manuscript.

### Conflict of interest statement

The authors declare that the research was conducted in the absence of any commercial or financial relationships that could be construed as a potential conflict of interest.
