# Supplementary material for: Donor NKG2C Copy Number: An Independent Predictor for CMV Reactivation After Double Cord Blood Transplantation
Source: Front Immunol. 2018 Oct 23;9:2444. doi: 10.3389/fimmu.2018.02444 (PMC6206267; doi:10.3389/fimmu.2018.02444)
Supplement: Supplementary file 1 [file Data_Sheet_1.docx]

**Supplementary Materials**

**Supplementary Figure 1**- NKG2C genotyping. Upper band of 411 bp fragment is observed only in carriers of NKG2C deletion (del), and the lower band of 201 bp only in carriers of NKG2C gene (wt). From left to right, lane 1 – lane 5, 5 DNA samples of donors with different genotypes: wt/del, del/del, wt/wt, wt/del, wt/wt, respectively; lane 6, homozygote control for NKG2C (wt/wt); lane 7, heterozygote control (wt/del); lane 8, homozygote control for NKG2C deletion (del/del); lane 9, H_2_O contamination control; and lane 10, molecular weight marker, 150 bp ladder


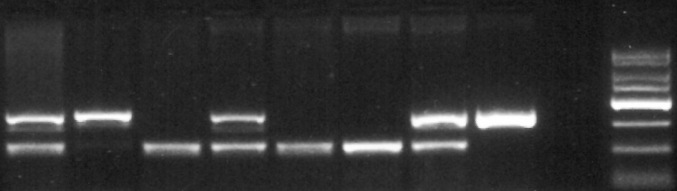


**1**

**wt/del**

**2**

**del/del**

**3**

**wt/wt**

**4**

**wt/del**

**5**

**wt/wt**

**6**

**wt/wt**

**7**

**wt/del**

**8**

**del/del**

**9**

**H_2_O**

**Size Marker**

201 bp (wt)

411 bp (del)
